# A Review of the Feather Mite Genus *Lopharalichus* Gaud & Atyeo, 1996 (Acariformes: Pterolichidae), with Descriptions of Three New Species from Brazilian Parrots (Psittaciformes: Psittacidae) †

**DOI:** 10.3390/ani13142360

**Published:** 2023-07-19

**Authors:** Fabio Akashi Hernandes

**Affiliations:** Departamento de Ecologia e Zoologia (ECZ), Centro de Ciências Biológicas, Federal University of Santa Catarina, Florianópolis 88040-970, Brazil; abakashi@gmail.com

**Keywords:** avian mites, diversity, taxonomy, systematics, Psoroptidia, Pterolichoidea

## Abstract

**Simple Summary:**

Understanding the current biodiversity of our planet is an ongoing challenge, as natural habitats are being destroyed at a faster rate than species are described. This is especially true for South America, which harbors over one-third of the parrot species in the world. A diverse yet poorly studied group of mites associated with birds are feather mites, which currently include about 2500 known species, and estimates range from 10,000 to 20,000 species. Herein, three new species of feather mites of the genus *Lopharalichus* are described from parrots in Brazil.

**Abstract:**

Feather mites of the genus *Lopharalichus* Gaud & Atyeo, 1996 (Pterolichidae: Pterolichinae), formerly containing three described species, are associated with New World parrots (Psittaciformes: Psittacidae) of the subfamily Arinae. Three new species of this genus are described: *Lopharalichus tuim* **sp. nov.** from *Forpus xanthopterygius* (Spix, 1824), *L. spinosus* **sp. nov.** from *Ara ararauna* (Linnaeus, 1758), and *L. chiriri* **sp. nov.** from *Brotogeris chiriri* (Vieillot, 1818). Type specimens of the previously described *Lopharalichus* species were examined, and a key to the known species is provided.

## 1. Introduction

Three groups of feather mite genera from the subfamily Pterolichinae (Acariformes: Pterolichidae) are found on parrots (Psittaciformes): *Protolichus*, *Psittophagus*, and *Rhytidelasma* groups [1,2,3]. The *Protolichus* generic group, incorporating nearly 100 described species in 24 genera, is the most diverse of these groups, with 11 genera found on parrots of the New World [4]. The genus *Lopharalichus* Gaud & Atyeo, 1996 belongs to this group and has included, to date, three species [5,6]: *Lopharalichus denticulatus* (Mégnin & Trouessart, 1884) from *Pyrrhura cruentata* (Wied-Neuwied, 1820) from Brazil, *L. cribiformis* (Mégnin & Trouessart, 1884) from *Forpus passerinus* (Linnaeus, 1758) from Guyana, and *L. beckeri* Mironov, Dabert & Ehrnsberger, 2005 from *Conuropsis carolinensis* (Linnaeus, 1758), an extinct parrot of North America. Gaud & Atyeo [5] presented illustrations of two undescribed species from two other New World parrots, *Thectocercus acuticaudatus* (Vieillot, 1818) (formerly *Aratinga acuticaudata*) and *Forpus modestus sclateri* (Gray, 1859) (formerly *Forpus sclateri*). An undetermined *Lopharalichus* species was reported from *Brotogeris chiriri* (Vieillot, 1818) [7] (not confirmed whether it corresponds to the new species described herein form the same parrot species). Pedroso and Hernandes [8] reported three undescribed species of *Lopharalichus* from Brazil, and these mites are described below.

The most distinctive feature of the genus *Lopharalichus* is the presence of prominent spiny crests on the femora and genua of legs I and II of both males and females, after which the genus was named (Gr. *lophos* = crest, mane). Other noticeable features are as follows: in both sexes, the lateral regions of hysterosoma have small cuticular spines, setae *c*2 are bifid, scapular setae *si* are subequal to or longer than setae *se*, setae *se* are very short (at maximum ½ the distance *si:se*), the prodorsal shield is entire (unlike species of the genera *Aralichus* Gaud 1966, *Chelomatolichus* Gaud & Atyeo 1996, and *Pararalichus* Atyeo 1989, in which the shield is divided by a transverse band of weakly sclerotized area at level of scapular setae *si*, *se*), and setae *h*1 are absent. Additionally, in males, setae *h*2 and *h*3 are flatly expanded with a filamentous tip, setae *e*2 are bifid with a short basal spine (except in *L. denticulatus*), setae *f*2 are expanded (leaf-like), setae *ps*1 are broad, and in females, setae *e*2 and *ps*1 are short, expanded with minute spines.

In this paper, three new species of *Lopharalichus* are described from parrots of Brazil, and a key to the known species of this genus is presented.

## 2. Materials and Methods

The new mites studied herein were collected from either wild bird specimens found dead in the field or from taxidermied bird specimens (see below). In the former case, the birds were collected and frozen for a later study; in laboratory, they were washed in a plastic tray with water and detergent to remove the ectoparasites [9], and the water was filtered through a paper filter. The mites were collected from the filters with a fine brush under a dissecting microscope. A few specimens from *Ara ararauna* (Linnaeus) were also retrieved from dry museum skins deposited at the Museu de História Natural Capão da Imbuia (MHNCI), Curitiba, following the ruffling technique described in Gaud & Atyeo [5]. The mites obtained with both methods were cleared and distended in 30% lactic acid at 50 °C for 24 h, mounted on microscopic slides using Hoyer’s medium [10], and heated and dried at 50 °C for 5 days. Finally, the edges of the coverslips were sealed with transparent varnish and the slides were labeled. The specimens were studied under an Olympus CX31 microscope, and illustrations were prepared from pictures of the mites taken with a digital camera (Omax A35140U 14mpx, Chengdu, China) attached to the ocular lenses and produced on Adobe Illustrator CS5 using a Wacom Bamboo Create tablet. The chaetotaxies of idiosoma and legs follow Griffiths et al. [11] and Atyeo & Gaud [12], respectively, with further corrections for coxal setae [13]. The nomenclature of birds is according to Gill et al. [14].

The species descriptions are given according to the formats proposed by Mironov et al. [6] and Hernandes [4]. Type specimens of the new species are deposited at the Acari Collection of the Department of Ecology and Zoology of the Universidade Federal de Santa Catarina, Florianópolis (ECZ–UFSC). Additional material examined consisted of types and other specimens of *Lopharalichus cribriformis* and *L. denticulatus* determined by W.T. Atyeo and are deposited at the Trouessart collection of the Muséum National d’Histoire Naturelle (MNHN), Paris, France. Photos of non-type specimens of *L. beckeri* deposited at the Zoology Institute, Russian Academy of Sciences (ZISP), St. Petersburg, were also examined.

## 3. Results

### Systematics

Pterolichidae Trouessart & Mégnin, 1884

*Lopharalichus* Gaud & Atyeo, 1996

(*Lopholichus*, Gaud & Atyeo 1996:121, *sic*)

Type species: *Pterolichus* (*Pterolichus*) *denticulatus* Mégnin & Trouessart, 1884, by original designation.

**Figure 1:**
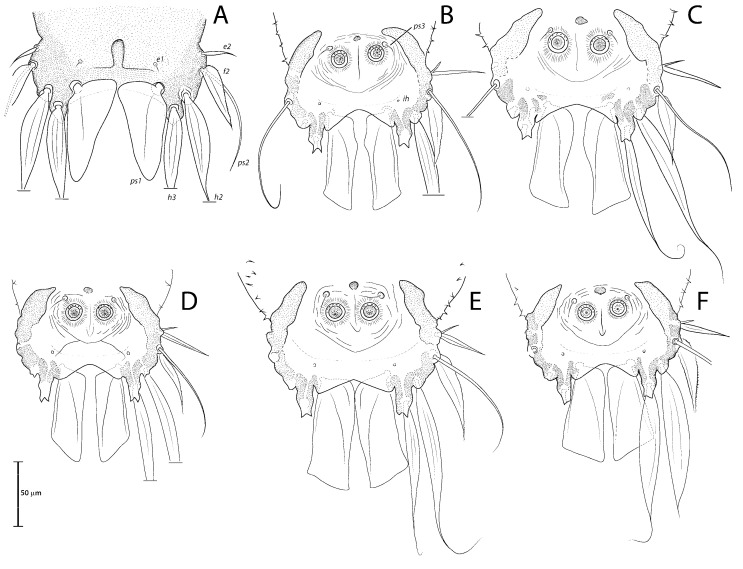
Opisthosoma of *Lopharalichus* spp. males (A = dorsal; B–F = ventral): *L. denticulatus* (**A**); *L. cribriformis* (**B**); *L. beckeri* (**C**,); *L. tuim* sp. nov. (**D**); *L. spinosus* sp. nov. (**E**); *L. chiriri* sp. nov. (**F**).

**Figure 2:**
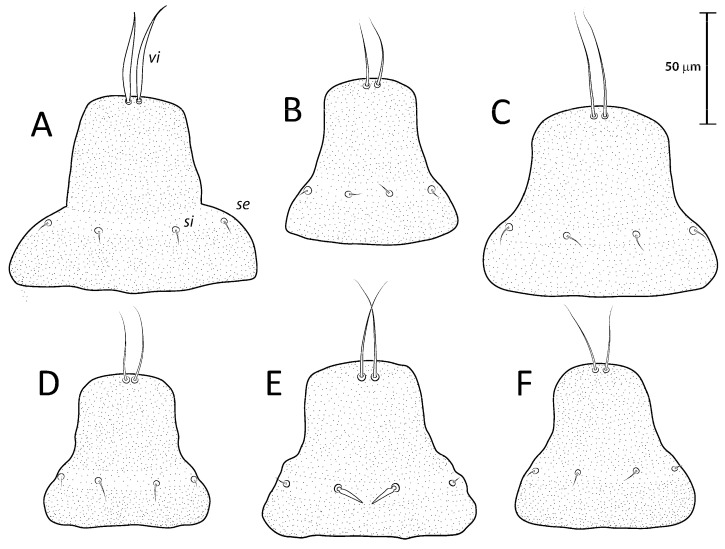
Prodorsal shield of *Lopharalichus* spp. males: *L. denticulatus* (**A**); *L. cribriformis* (**B**); *L. beckeri* (**C**); *L. tuim* sp. nov. (**D**); *L. spinosus* sp. nov. (**E**); *L. chiriri* sp. nov. (**F**).

**Figure 3:**
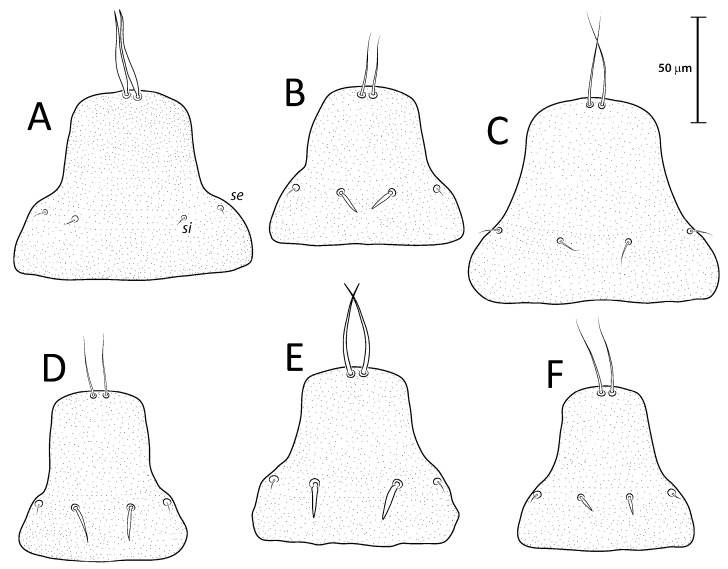
Prodorsal shield of *Lopharalichus* spp. females: *L. denticulatus* (**A**); *L. cribriformis* (**B**); *L. beckeri* (**C**); *L. tuim* sp. nov. (**D**); *L. spinosus* sp. nov. (**E**); *L. chiriri* sp. nov. (**F**).

**Figure 4:**
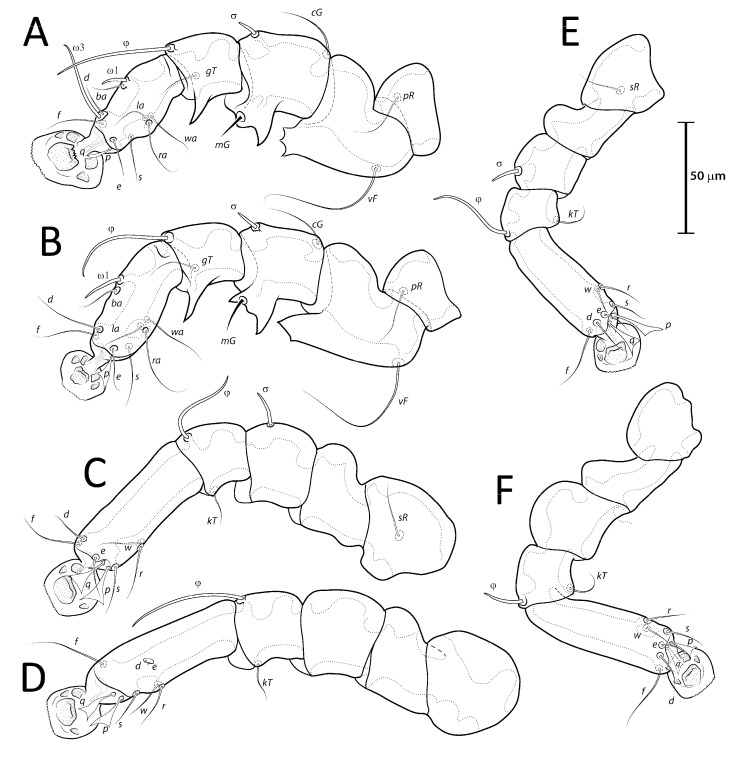
*Lopharalichus denticulatus*, legs I–IV (**A**–**D**) of male; legs III–IV (**E**,**F**) of female.

***Lopharalichus denticulatus* (Mégnin & Trouessart, 1884)**

(Figure 1A, Figure 2A, Figure 3A and Figure 4)

*Pterolichus* (*Pterolichus*) *denticulatus* Mégnin & Trouessart, 1884 [15]: 211.

*Pterolichus* (*Eupterolichus*) *denticulatus*; Canestrini & Kramer, 1899 [16]: 37.

*Pterolichus denticulatus*; Radford, 1953 [17]: 201; Gaud & Atyeo, 1996 [5]: 128.

Type material examined: Lectotype male ex *Pyrrhura cruentata* (Wied-Neuwied, 1820) (Psittaciformes: Psittacidae) from BRAZIL, no further data, MNHN#969.236.3 (slide 35-I-6) (the remounted slide also contains a paralectotype male of *Neorhytidelasma tritiventris* (Trouessart, 1884)).

Additional material examined: One male ex *P. cruentata*, BRAZIL, Bahia state, Boa Nova, 5 June 1928, E. Kaempfer (AMNH241747, UGA10,450), MNHN#1060.31.2 (slide 65-D-6) (W.T. Atyeo det. 1993); one female ex *P. cruentata*, BRAZIL, Espírito Santo state, Lagoa Juparanã, 11 November 1929, E. Kaempfer (AMNH317283, UGA10,452), MNHN#1060.31.1 (slide 65-D-5) (W.T. Atyeo det. 1993).

Remarks: *Lopharalichus denticulatus* stands out from other species in having, in males, setae *ps*1 roughly triangular, setae *e*2 simple and not bifurcate basally; in females, the prodorsal setal pair *si* is well spaced by about three-times the distance *si:se* (Figure 3A); in both sexes, vertical setae *vi* are slightly expanded (Figure 2A and Figure 3A), genua I, II have prominent, thick antiaxial crests, and the hysteronotal shield is usually devoid of lacunae; in one non-type male examined, there are a few small, sparse circular lacunae in the center of the shield, about 1–3 μm in diameter. The only examined female is broken, with legs, epigynum, and other structures displaced from their original position.

**Figure 5:**
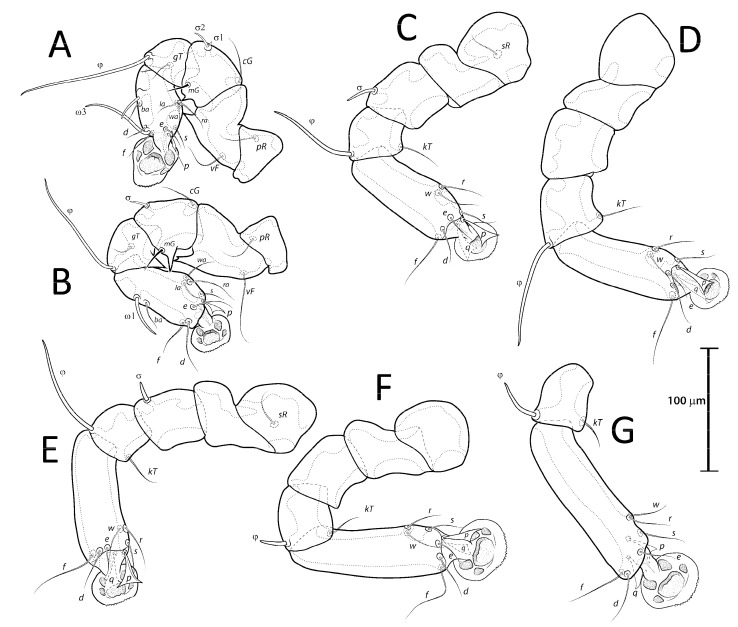
*Lopharalichus cribriformis*, legs I–IV (**A**–**D**) of male; legs III–IV (**E**,**F**) of female. Lopharalichus beckeri, tibia, and tarsus IV of female (**G**).

***Lopharalichus cribriformis* (Mégnin & Trouessart, 1884)**

(Figure 1B, Figure 2B, Figure 3B and Figure 5A–F)

*Pterolichus* (*Pterolichus*) *denticulatus* var. *cribriformis* Mégnin & Trouessart, 1884 [15]: 213.

*Pterolichus* (*Eupterolichus*) *cribriformis*; Canestrini & Kramer, 1899 [16]: 38.

*Pterolichus denticulatus*; Radford, 1953 [17]: 201.

Type material examined: Syntypes 15 males and 19 females (in the same original slide, not remounted) ex *Forpus passerinus* (Linnaeus, 1758) (=*Psittaculus passerinus*), from GUYANA, MNHN#969.237.1 (slide 35-I-8).

Additional material examined: one male and one female ex *Forpus passerinus cyanochlorus* (Schlegel, 1864), BRAZIL, Amazonas state, Frechal, Rio Surumu, 6 September 1929, T.D. Carter col. (AMNH236355, UGA12,742) MNHN#1060.30 (slide 65-D-4) (W.T. Atyeo det. 1993).

Remarks: *Lopharalichus cribriformis* is very similar to *L. beckeri* Mironov et al. (2005), differing from that species in having, in males, the terminal cleft angular and the paragenital apodemes indistinctly developed, and in females, setae *si* distinctly longer and more robust than *se* (at least twice longer and twice thicker) (Figure 3B), and the solenidion on tibia IV as long as half the width of this segment (Figure 5F). In males of *L. beckeri*, the lobar cleft is nearly semicircular (Figure 1C), and the paragenital apodemes are distinctly formed; and in females, setae *se* and *si* are both piliform and similar in structure (Figure 3C), and solenidion φ on tibia IV is about the same length as the width of tibia (Figure 5G).

Mironov et al. [6] stated that, in males of *L. cribriformis*, setae *e*2 are twice as long as *f*2, and in females, setae *f*2 are “large and foliform, almost circular, and with a vein”. However, in the examined specimens of this species, setae *e*2 and *f*2 have about the same length in males, and setae *f*2 of females are roughly triangular, like in *L. beckeri*. The type series of *L. cribriformis* consists of a single slide containing 34 poorly clarified syntypes, still with the original label by E.L. Trouessart. The illustrations presented here are based on non-type material collected from the type host species and determined by W.T. Atyeo.

***Lopharalichus beckeri* Mironov, Dabert & Ehrnsberger, 2005**

Figure 1C, Figure 2C, Figure 3C and Figure 5G)

*Lopharalichus beckeri* Mironov, Dabert & Ehrnsberger, 2005 [5]: 2259

Material examined: Photos of 1 male and 1 female (ZISP 6760, 6767) ex *Conuropsis carolinensis* (MCZ 209911, UNAM 110), USA, Florida, Tampa, no date, coll. W. Brewster.

Remarks: *Lopharalichus beckeri* was described from *Conuropsis carolinensis* (Linnaeus, 1758), an extinct parrot from North America. This species is very similar to *L. cribriformis* (see differential characters in the remarks of the previous species).

**Figure 6:**
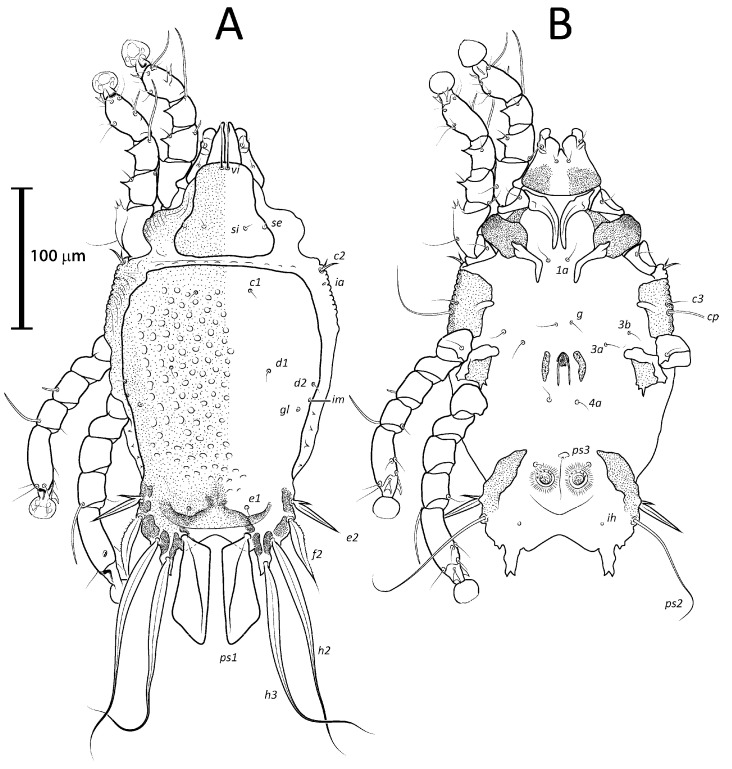
*Lopharalichus tuim* sp. nov. male: dorsal (**A**) and ventral (**B**) views.

**Figure 7:**
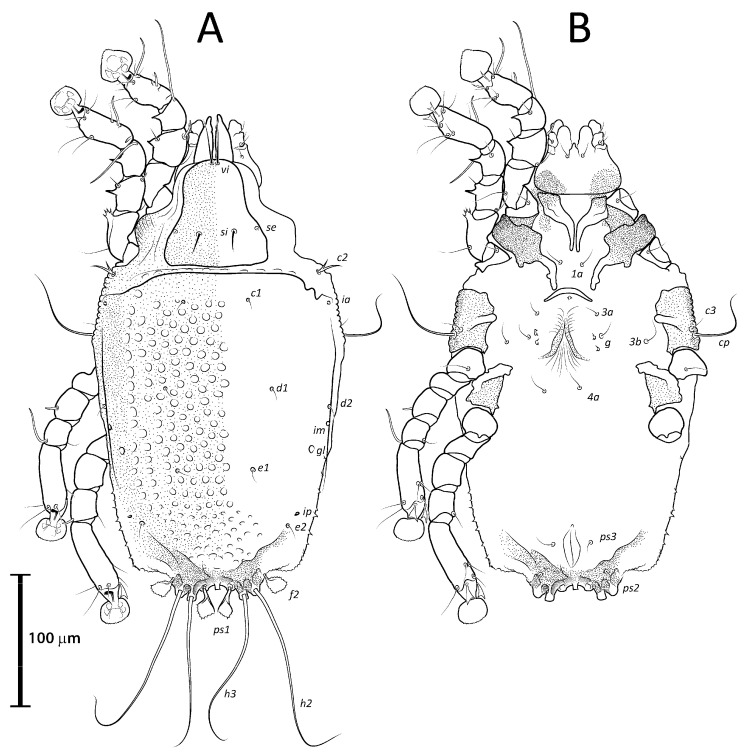
*Lopharalichus tuim* sp. nov. female: dorsal (**A**) and ventral (**B**) views.

**Figure 8:**
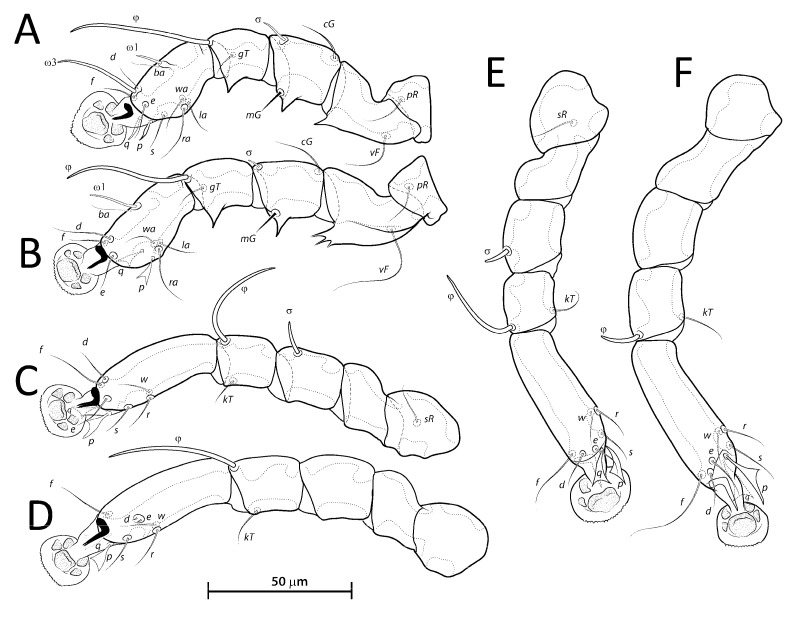
*Lopharalichus tuim* sp. nov., legs I–IV (**A**–**D**) of male; legs III–IV (**E**,**F**) of female.

***Lopharalichus tuim* sp. nov.**

(Figure 1D, Figure 2D, Figure 3D and Figure 6, Figure 7 and Figure 8)

Zoobank registration: urn:lsid:zoobank.org:act:7BAEC958-A174-42FC-8C28-0198480DC854

Type material. Holotype male, paratypes 10 males, 31 females, and 1 nymph ex *Forpus xanthopterygius* (Spix, 1824) (Psittaciformes: Psittacidae), BRAZIL, São Paulo State, Pedreira, 22°44′ S, 46°54′ W, June 2012, D.V. Boas-Filho col. (#1074).

Male (holotype, range for five paratypes in parentheses). Idiosoma length (from the level of setae *vi* to the base of setae *h*3) 284 (284–297), greatest width at level of humeral shields 162 (163–176). Prodorsal shield shaped as an Erlenmeyer flask (elongated trapezoid), with rounded edges, posterior margin slightly sinuous, surface without ornamentation, 64 (64–72) in length from the level of setae *vi* to the posterior margin, 69 (69–78) in width at the widest part. Scapular setae *si* thin spiculiform, 9 (8–11) long, setae *se* piliform, reduced, distance between bases of scapular setae *si:si* 29 (25–27), *se:se* 57 (56–61). Hysterosoma 212 (211–226) in length from sejugal area to the bases of setae *h*3. Hysteronotal shield: anterior margin straight, length from anterior margin to bases of setae *h*3 207 (212–227), greatest width at the level of setae *d*2 138 (144–158), surface with numerous circular lacunae posterior to level of setae *c*1 (Figure 6A), supranal concavity poorly distinct, anterior to level of setae *e*1. A bow-shaped transverse fold between levels of setae *e*1 and *ps*1. Membranous margin of terminal cleft (=contour of free margin of interlobar membrane) blunt-angular, 28 (30–34) long, opisthosomal lobes with prominent tubercles at bases of setae *h*3, narrow interlobar membrane between bases of setae *ps*1. Setae *c*2 bifid, 12 (12–15) long; setae *e*2 lanceolate with short basal bifurcation, 45 (42–49) long; setae *f*2 lanceolate with outer edge minutely serrate, 54 (56–65); setae *ps*1 roughly parallelogram-shaped, 78 (77–84) long. Distances between hysteronotal setae: *c*2*:d*2 72 (84–90), *d*2*:e*2 84 (76–82), *e*2*:h*3 40 (45–50), *d*1*:d*2 10 (8–13), *e*1*:e*2 4 (5–13), *ps*1*:ps*1 45 (43–51), *h*3*:h*3 69 (66–76), *h*2*:h*2 82 (82–92), and *ps*2*:ps*2 106 (106–117).

Bases of epimerites I and II with inflations and dark sclerotized (Figure 6B). Humeral shields developed ventrally and bearing setae *c*3, *cp*. Setae *c*3 thin piliform, 14 (12–16) long; coxal fields I–II without sclerotized areas. Genital apparatus situated between levels of trochanters III and IV, 24 (24–28) long, 11 (10–13) wide; paragenital apodemes as a pair of longitudinal sclerites lateral to the genital apparatus and bearing the genital acetabula. Distances between setae: *g:4a* 55 (51–58), *g:g* 8 (6–9). Cupules *ih* ventrally at the level of setae *ps*2. Adanal suckers 13 (13–15) in diameter, distance between centers of suckers 24 (22–26), corolla with 5–7 teeth on anterior half, posterior half without teeth (Figure 1D and Figure 6B).

Femora I with 1–3 apicoventral spines or crests, femur II with 2–6 apicoventral spines. Acute apicoventral spines on genua, tibiae I, II. Length of tarsi excluding ambulacra: tarsus I 37 (35–38), tarsus II 44 (46–50), tarsus III 49 (49–54), tarsus IV 55 (55–57). Seta *kT* present on tibia IV. Setae *d*, *e* minute spiculiform, inserted close together (Figure 8D). Setae *p*, *q* on tarsi I thinner and apically less expanded than on tarsi of other legs. Solenidion *σ*2 of genu I apparently absent. Length of solenidia: *σ*2*I* 10 (9–12), *σIII* 9 (7–9), *φI* 50 (50–55), *φII* 45 (43–47), *φIII* 33 (30–33), *φIV* 40 (35–43), *ω*1*I* 10 (10–12), *ω*3*I* 29 (29–32), and *ω*1*II* 19 (17–18).

Female (range for 6 paratypes). Idiosoma length 309–334, greatest width 173–185. Prodorsal shield-shaped as in the male, 73–79 long, 76–79 wide (Figure 7A); scapular setae *si* spiculiform, 14–17 long, setae *se* piliform, reduced; distances between scapular setae *si:si* 21–28, *se:se* 59–63. Hysteronotal shield 239–247 in length, 162–171 in width at the widest part; surface with numerous circular lacunae posterior to level of setae *c*1. Setae *c*2 bifid, setae *f*2, *ps*1 flat, spiky leaf-like, setae *c*1, *d*1, *d*2, *e*1, *e*2 piliform. Terminal region of opisthosoma shaped as a semicircular concavity between a pair of tubercles bearing setae *h*2, *h*3, and with a small external copulatory tube in the center about 5–7 long located between setae *ps*1. Posterolateral margins of opisthosoma with small spines. Length of setae: *c*2 12–15, *c*3 16–31, *e*2 11–16, and *f*2 16–22. Distances between dorsal setae: *c*2*:d*2 95–101, *d*2*:e*2 89–93, *d*1*:d*2 11–20, *e*1*:e*2 40–46, *ps*1*:ps*1 17–21, *h*3*:h*3 37–40, *h*2*:h*2 52–56.

Epimerites I free. Bases of epimerites I, II inflated, dark-sclerotized (Figure 7B). Epigynum as a low arch, 9–13 in length, 24–35 in width. Distance between ventral setae 1*a:*3*a* 36–42, 3*a:g* 20–30. Legs I, II as in the male, except for a shorter apicoventral spine on genu and tibia I. Length of tarsi excluding ambulacra: tarsus I 35–42, tarsus II 44–50, tarsus III 53–56, tarsus IV 59–65. Length of solenidia: *σ*2*I* 10–13, *σIII* 9–10, *φI* 61–62, *φII* 50–55, *φIII* 35–38, *φIV* 12–16, *ω*1*I* 11–13, *ω*3*I* 30–34, and *ω*1*II* 18–25.

Differential diagnosis: The new species, *L. tuim* sp. nov., is very close to *L. cribriformis* (Mégnin & Trouessart, 1884) described from *Forpus passerinus* in having a blunt-angular terminal cleft in males. In males of *L. denticulatus* and *L. beckeri,* the lobar cleft is concave and semi-circular. The new species most clearly differs from *L. cribriformis* in the relative length and arrangement of prodorsal setae *si*: in females of *L. tuim sp.* nov., *si* reaches the base of *se* of the same side (Figure 3D), and in males, *si* reaches at least halfway to the base of corresponding setae *se*, *si* being about twice longer than *se* (Figure 2D). Also, in males of the new species, setae *si* are inserted slightly closer to the corresponding *se* than to the other member of the pair *si* (distance *si:si* is about 1 ½ the distance *si:se*). In *L. cribriformis* females, setae *si* only reach about halfway to the bases of corresponding *se* (Figure 3B), and in males of that species, these setae reach one-third of that distance (*si* is about the same length as *se*) (Figure 2B), and in both sexes, the scapular setae *si* and *se* are uniformly spaced (distance *si:se* = *si:si*).

Etymology: The name of the new species is based on the Brazilian common name of the host (tuim) and is a noun in apposition.

**Figure 9:**
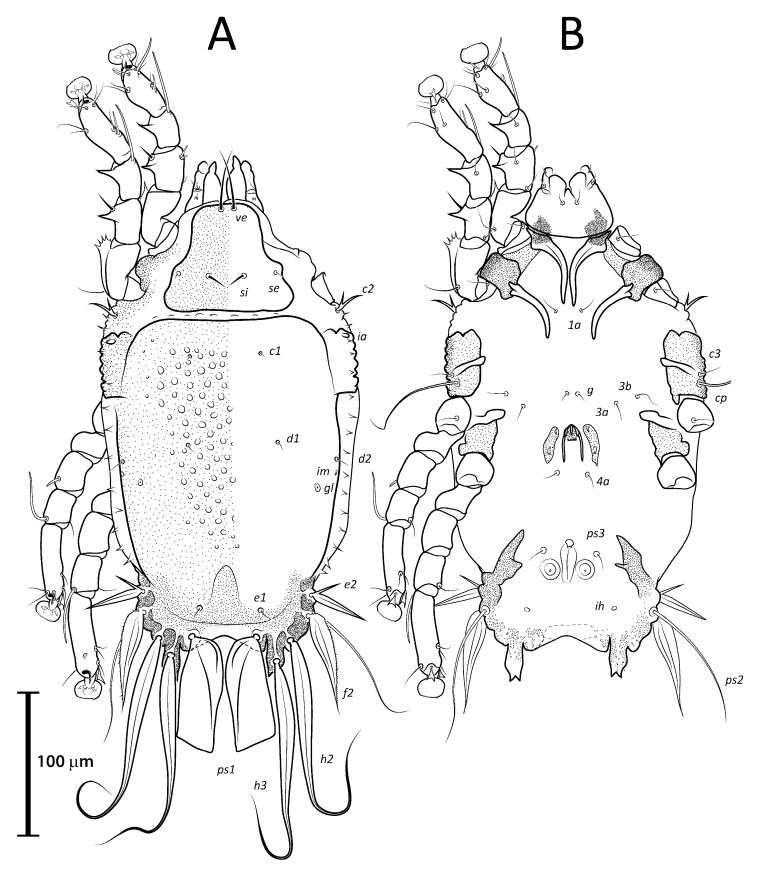
*Lopharalichus spinosus* sp. nov. male: dorsal (**A**) and ventral (**B**) views.

**Figure 10:**
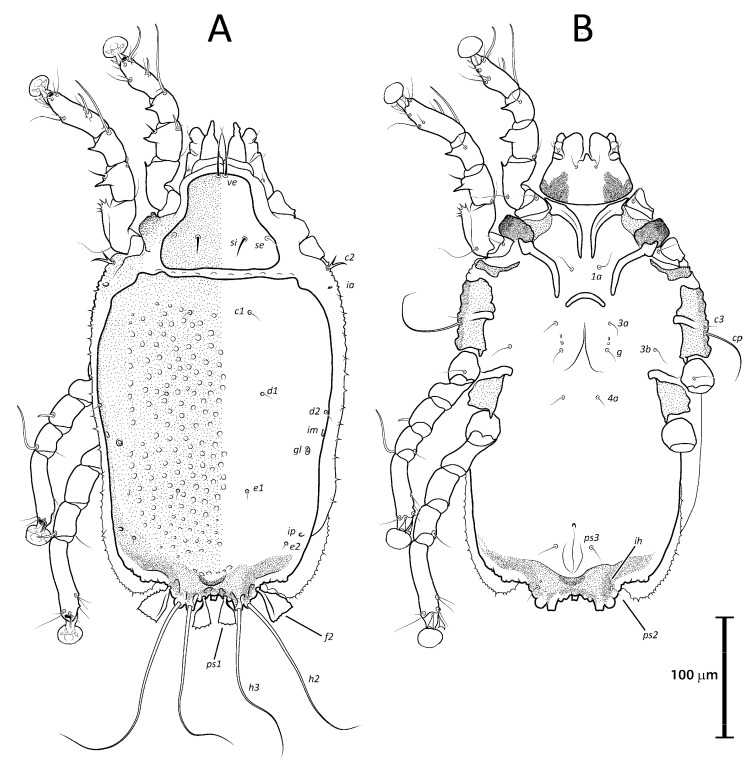
*Lopharalichus spinosus* sp. nov. female: dorsal (**A**) and ventral (**B**) views.

**Figure 11:**
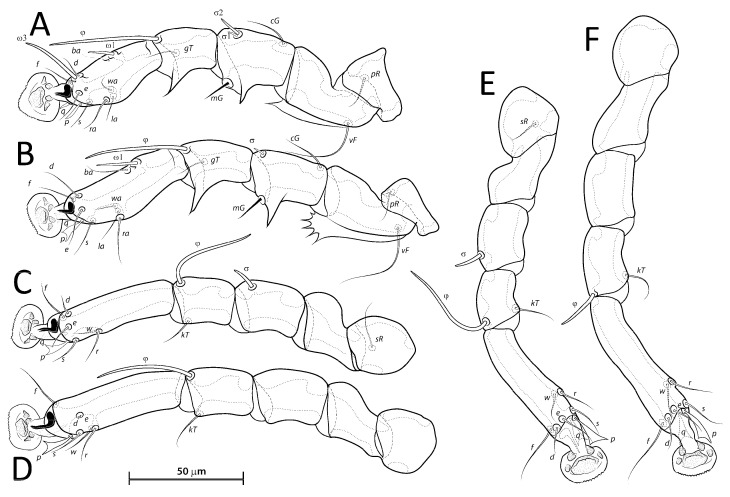
*Lopharalichus spinosus* sp. nov., legs I–IV (**A**–**D**) of male; legs III–IV (**E**,**F**) of female.

***Lopharalichus spinosus* sp. nov.**

(Figure 1E, Figure 2E, Figure 3E and Figure 9, Figure 10 and Figure 11)

Zoobank registration: urn:lsid:zoobank.org:act:12DC2DDA-DE5D-4356-AF19-53F77CB37A96

Type material: holotype male, seven male and six female paratypes ex *Ara ararauna* (Linnaeus, 1758) (Psittaciformes: Psittacidae), BRAZIL, São Paulo State, Itatiba, 23°00′ S, 46°50′ W, 24 March 2007, U. Kawazoe col. (#152). Paratypes from the same host species: five males and four females, Pernambuco State, 29 September 1953 (MHNCI#1557), mites collected from the bird skin by FAH in November 2016.

Male (holotype, range for two paratypes in parentheses). Idiosoma length from the level of setae *vi* to the base of setae *h*3 345 (338–350), greatest width at level of humeral shields 190 (190–193). Prodorsal shield shaped roughly as an isosceles trapezoid, with sinuous lateral margins and rounded edges, 78 (76–78) in length from the level of setae *vi* to the posterior margin, 96 (93–96) in width at the posterior margin. Scapular setae *si* as a short spike, about as long as the distance between their bases, 13 (11–13) long, distance between scapular setae *si:si* 25 (24–25), *se:se* 75 (73–77). Hysterosomal region 263 (256–265) in length from sejugal area to the bases of setae *h*3. Hysteronotal shield: anterior margin straight, length from anterior margin to bases of setae *h*3 260 (254–256), greatest width around the level of setae *d*2 165 (167–176), surface with numerous circular lacunae from the level of setae *c*1 to genua IV (Figure 9A). A bow-shaped transverse fold between levels of setae *e*1 and *ps*1. Membranous margin of terminal cleft blunt-angular, 30 (30–30) long, opisthosomal lobes with prominent tubercles at bases of setae *h*3, narrow interlobar membrane between bases of setae *ps*1. Setae *c*2 bifid, 21 (17–21) long; setae *e*2 lanceolate with short basal bifurcation, greatest length 43 (40–43); setae *f*2 lanceolate with external margin minutely serrate, 66 (64–66); setae *ps*1 roughly parallelogram-shaped with sharp posterior edges, 89 (88–90) long. Distances between hysteronotal setae: *c*2*:d*2 111 (106–111), *d*2*:e*2 101 (95–98), *e*2*:h*3 52 (50–52), *d*1*:d*2 11 (13–14), *e*1*:e*2 11 (9–11), *ps*1*:ps*1 49 (45–52), *h*3*:h*3 78 (75–78), *h*2*:h*2 96 (92–96), and *ps*2*:ps*2 126 (116–126).

Bases of epimerites I, II inflated, dark-sclerotized (Figure 9B). Humeral shields bearing setae *c*3, *cp* ventrally. Setae *c*3 thin piliform, 19 (17–19) long, coxal fields I, II without sclerotized areas. Genital apparatus situated between levels of trochanters III, IV, 30 (27–30) long, 12 (12–14) wide; paragenital apodemes as a pair of longitudinal sclerites roughly parallel to the arms of genital arch and bearing genital acetabula. Distances between setae: *g:4a* 66 (61–67), *g:g* 9 (7–9). Cupules *ih* ventrally at the level of setae *ps*2. Adanal suckers 15 (14–17) in diameter, distance between centers of suckers 27 (25–27), corolla with 5–7 teeth on anterior half, posterior half without teeth.

Femora I, II with 3–5 apicoventral spines or crests. Acute apicoventral spines on genua, tibiae I, II. Length of tarsi excluding ambulacra: tarsus I 44 (40–42), tarsus II 55 (53–55), tarsus III 57 (58–60), tarsus IV 67 (62–65). Seta *kT* present on tibia IV. Setae *d*, *e* minute spiculiform inserted together (Figure 11D). Genual solenidion *σ*1 on genu I present, minute, about 5 in length. Length of solenidia: *σ*2*I* 10 (10–11), *σIII* 10 (8–10), *φI* 49 (46–50), *φII* 43 (42–44), *φIII* 47 (39–42), *φIV* 45 (37–42), *ω*1*I* 11 (10–11), *ω*3*I* 30 (26–30), and *ω*1*II* 20 (17–20).

Female (range for six paratypes). Idiosoma length 367–399, greatest width 197–212. Prodorsal shield shaped as in the male, 82–90 long, 94–106 wide (Figure 10A); scapular setae *si* spiculiform, 17–20 long, setae *se* piliform; distances between scapular setae *si:si* 29–39, *se:se* 78–88. Hysteronotal shield 278–307 in length, 179–190 in width at the widest part at the level of setae *d*1; surface with numerous circular lacunae from the level between setae *c*1 to *e*2. Lateral hysterosomal setae *c*2 bifid, *c*1, *d*1, *d*2, *e*1, *e*2 thin piliform, setae *f*2, *ps*1 flat, spiky leaf-like. Terminal region of opisthosoma shaped as a semicircular concavity flanked by a pair of tubercles bearing setae *h*2 and *h*3. Posterolateral margins of opisthosoma with small spines. Terminal margin of opisthosoma between setae *ps*1 with small copulatory extension about 5–7 long. Length of setae: *c*2 14–20, *e*2 10–14, *c*3 20–29, and *f*2 23–32. Distances between dorsal setae: *c*2*:d*2 113–124, *d*2*:e*2 110–121, *d*1*:d*2 7–13, *e*1*:e*2 39–48, *ps*1*:ps*1 21–26, *h*3*:h*3 42–49, and *h*2*:h*2 59–65.

Epimerites I free, bases of epimerites I, II inflated, dark-sclerotized (Figure 10B). Epigynum as a low arch, 12–18 in length, 34–46 in width. Distance between ventral setae 1*a:*3*a* 46–55, 3*a:g* 14–24. Legs I, II as in the male. Length of tarsi excluding ambulacra: tarsus I 39–47, tarsus II 56–61, tarsus III 61–66, tarsus IV 70–76. Solenidion *σ*1*I* present, minute, about 5 in length. Length of solenidia: *σ*2*I* 10–13, *σIII* 11–13, *φI* 56–64, *φII* 51–60, *φIII* 41–58, *φIV* 13–18, *ω*1*I* 10–14, *ω*3*I* 27–34, *ω*1*II* 19–21.

Differential diagnosis: *Lopharalichus spinosus* **sp. nov.** is close to *L. beckeri* and *L. cribriformis* in having, in males, well-formed cuticular spines in the lateral part of idiosomal anterior to setae *e*2. In both sexes of *L. spinosus*, however, those spines are much more numerous and occupy a larger area, from the level of setae *cp* to that of setae *e*2; in addition, in males of the new species, scapular setae *si* are spiculiform, noticeably more robust than *se* (Figure 2E). In both sexes of *L. beckeri* and *L. cribriformis*, the lateral spines are present only from the level of trochanter IV to the level of setae *e*2. In males of *L. cribriformis* and *L. beckeri*, and in females of the latter species, both scapular setae *si* and *se* are thin piliform (Figure 2B,C and Figure 3C); in females of *L. cribriformis*, setae *si* are more robust than *se*, but they only reach halfway to the distance between those setae (Figure 3B), whereas in *L. spinosus* sp. nov. females, *si* reaches the bases of corresponding setae *se* (Figure 3E).

Etymology: the specific name is an adjective (masculine) referring to the numerous cuticular spines on the lateral margins of hysterosoma, more pronounced and numerous than in other known species.

**Figure 12:**
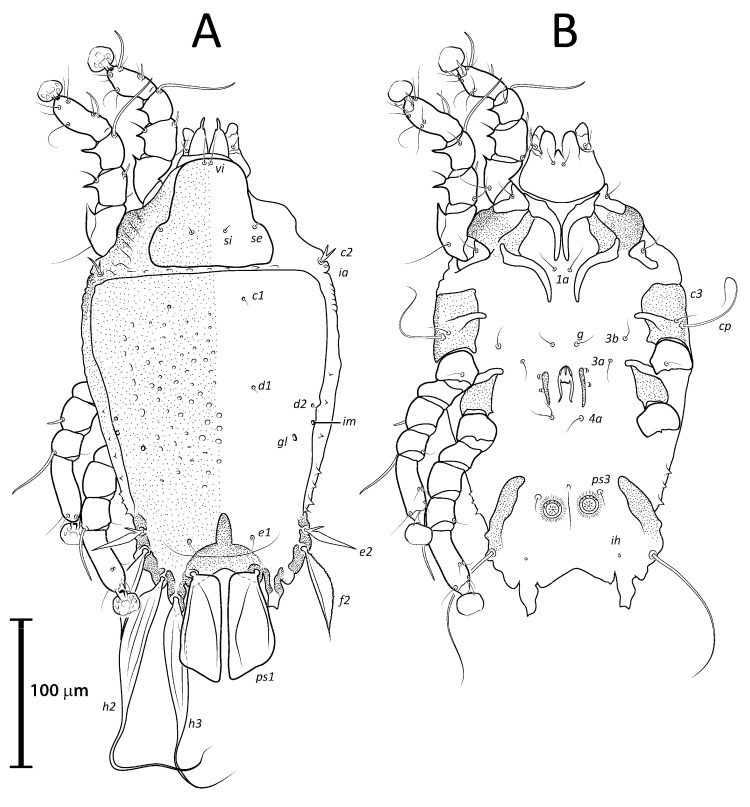
*Lopharalichus chiriri* sp. nov. male: dorsal (**A**) and ventral (**B**) views.

**Figure 13:**
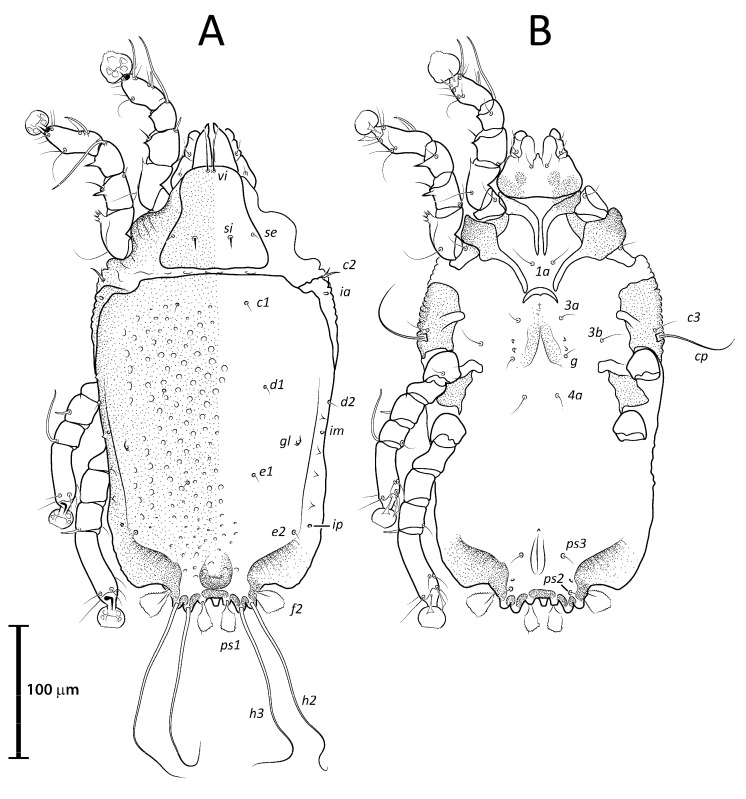
*Lopharalichus chiriri* sp. nov. female: dorsal (**A**) and ventral (**B**) views.

**Figure 14:**
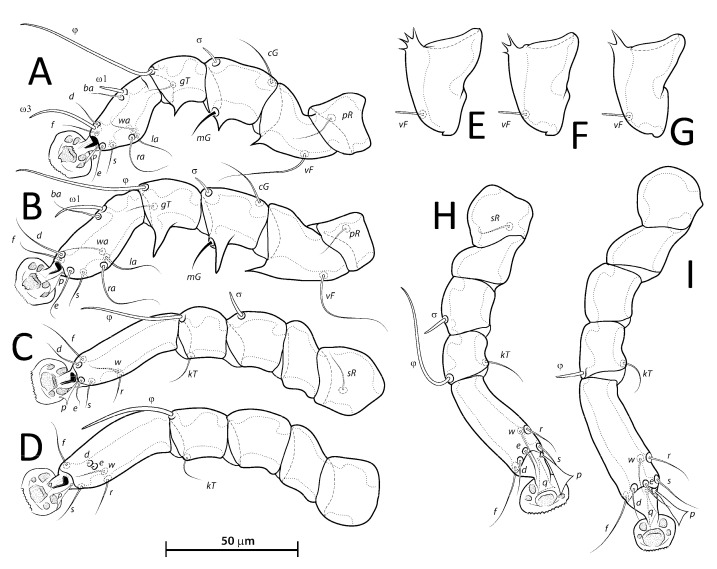
*Lopharalichus chiriri* sp. nov., legs I–IV (**A**–**D**) of male; variation in femora I in males (**E**–**G**); legs III–IV (**H**,**I**) of female.

***Lopharalichus chiriri* sp. nov.**

(Figure 1F, Figure 2F, Figure 3F and Figure 12, Figure 13 and Figure 14)

Zoobank registration: urn:lsid:zoobank.org:act:6DD91CE5-499F-43AD-8210-7B84DF879959

Type material: holotype male, 15 male and 8 female paratypes ex *Brotogeris chiriri*

(Vieillot, 1818) (Psittaciformes: Psittacidae), BRAZIL, São Paulo State, Pedreira, 22°44′ S, 46°54′ W, October 2013, D.V. Boas Filho col. (#1113); paratypes 4 females and 1 nymph, same host species, Pará State, Santana do Araguaia, Fazenda Fartura, 09°40′ S/50°23′ W, 07 September 2011, D.V. Boas-Filho coll. (#1006).

Male (holotype, range for six paratypes in parentheses). Idiosoma length from the level of setae *vi* to the base of setae *h*3 285 (294–308), greatest width at level of humeral shields 160 (160–167). Prodorsal shield roughly as an isosceles trapezoid with rounded posterior corners, 76 (67–74) in length from the level of setae *vi* to the posterior margin, 74 (76–79) in width at the widest part. Scapular setae *si* piliform, 7 (6–7) long, distance between *si:si* 23 (23–26), *se:se* 59 (57–62), *si:se* 17 (17–19). Hysterosomal region 224 (213–219) in length from sejugal area to the bases of setae *h*3. Hysteronotal shield: anterior margin straight, length from anterior margin to bases of setae *h*3 207 (213–219), greatest width around the level of setae *d*2 150 (140–155), surface with sparse circular lacunae from the level of setae *c*1 to genua IV (Figure 12A). A bow-shaped transverse fold between levels of setae *e*1 and *ps*1. Membranous margins of terminal cleft blunt-angular, 30 (28–31) long, opisthosomal lobes with prominent tubercles at bases of setae *h*3, and narrow interlobar membranes between bases of setae *ps*1. Setae *c*2 bifid, 12 (11–15) long; setae *e*2 lanceolate with short basal bifurcation, greatest length 35 (35–44); setae *f*2 lanceolate with outer margin minutely serrate, 54 (59–68); setae *ps*1 roughly parallelogram-shaped, 73 (73–82) long. Distances between hysteronotal setae: *c*2*:d*2 95 (94–100), *d*2*:e*2 81 (71–81), *e*2*:h*3 42 (41–50), *d*1*:d*2 16 (9–16), *e*1*:e*2 9 (8–14), *ps*1*:ps*1 42 (42–47), *h*3*:h*3 66 (67–72), *h*2*:h*2 82 (85–92), *ps*2*:ps*2 106 (107–115).

Bases of epimerites I, II inflated, dark-sclerotized (Figure 12B). Humeral shields bearing setae *c*3, *cp* ventrally. Setae *c*3 thin piliform, 17 (12–15) long, coxal fields I–II without sclerotized areas. Genital apparatus situated between levels of trochanters III and IV, 13 (10–13) long, 11 (10–11) wide; paragenital apodemes as a pair of thin longitudinal sclerites roughly parallel to the arms of genital arch and bearing genital acetabula. Distances between setae: *g:4a* 51 (47–53), *g:g* 9 (7–13). Cupules *ih* ventrally at the level of setae *ps*2. Adanal suckers 13 (13–15) in diameter, distance between centers of suckers 24 (22–27), corolla with 5–7 teeth on anterior half, posterior half without teeth.

Femora I, II with 1–4 apical spines on. Acute apicoventral spines on genua, tibiae I, II (slightly more developed on legs II than in legs I). Length of tarsi excluding ambulacra: tarsus I 33 (31–36), tarsus II 43 (40–45), tarsus III 46 (42–49), tarsus IV 48 (48–50). Seta *kT* present on tibia IV. Setae *d*, *e* minute spiculiform inserted close together. Solenidion *σ*2 of genu I apparently absent. Length of solenidia: *σ*2*I* 7 (7–9), *σIII* 9 (7–10), *φI* 50 (48–55), *φII* 43 (39–48), *φIII* 41 (34–45), *φIV* 40 (33–40), *ω*1*I* 12 (10–11), *ω*3*I* 26 (25–28), *ω*1*II* 19 (18–20).

Female (range for six paratypes). Idiosoma length 296–338, greatest width 171–189. Prodorsal shield shaped as an Erlenmeyer flask (elongated trapezoid), 68–80 long, 74–83 wide (Figure 13A); scapular setae *si* short spiculiform, 9–11 long, setae *se* piliform; distances between scapular setae *si:si* 21–28, *se:se* 59–65, *si:se* 16:21. Hysteronotal shield 233–254 in length, 163–174 in width at the widest part around level of setae *d*1; surface with numerous circular lacunae from the level between setae *c*1 to supranal concavity. Lateral hysterosomal setae *c*2 bifid, *c*1, *d*1, *d*2, *e*1, *e*2 thin piliform, setae *f*2, *ps*1 flat, spiky leaf-like. Terminal region of opisthosoma shaped as a semicircular concavity flanked by a pair of tubercles bearing setae *h*2, *h*3, and a small external copulatory tube around 5–7 in length between bases of setae *ps*1. Lateral margins of opisthosoma with few small spines. Length of setae: *c*2 9–13, *e*2 8–12, *c*3 14–17, *f*2 22–25. Distances between dorsal setae: *c*2*:d*2 99–112, *d*2*:e*2 85–101, *d*1*:d*2 10–20, *e*1*:e*2 31–46, *ps*1*:ps*1 16–20, *h*3*:h*3 35–41, *h*2*:h*2 53–57.

Epimerites I free, bases of epimerites I, II inflated, dark-sclerotized (Figure 13B). Epigynum as a low arch, 9–12 in length, 27–29 in width. Distance between ventral setae 1*a:*3*a* 37–54, 3*a:g* 17–21. Length of tarsi excluding ambulacra: tarsus I 30–37, tarsus II 40–46, tarsus III 42–48, tarsus IV 51–58. Length of solenidia: *σ*2*I* 8–11, *σIII* 7–11, *φI* 54–64, *φII* 48–58, *φIII* 38–47, *φIV* 10–14, *ω*1*I* 10–13, *ω*3*I* 24–29, *ω*1*II* 18–24.

Differential diagnosis: *Lopharalichus chiriri* sp. nov. is very similar to *L. cribriformis* due to the blunt-angular shape of terminal cleft in males but can be distinguished by the relatively longer distance between prodorsal setae *si-si*. In males of the new species, this distance is about 3.5-times the length of setae *si*, against 2.5-times that length in *L. cribriformis*. Also, the new species has smaller dorsal lacunae and relatively shorter solenidion on tibia IV in males, reaching only about half of the length of tarsus (it reaches at least ¾ of tarsus length in *L. cribriformis*). The new species is also distinguished from all previously known species in having, in both sexes, considerably longer solenidion on tibia III, roughly longer than the length of genu and tibia III combined. In females of *L. chiriri*, setae *si* are relatively shorter, their tips not touching each other (Figure 3F), while in *L. cribriformis* females, these setae do touch each other. Additionally, in both sexes of *L. chiriri*, tibial solenidion *φIII* is equal to the length of genu + tibia III (Figure 14C,H), while in other known species of *Lopharalichus*, solenidion *φIII* is shorter than the length of corresponding genu and tibia.

Etymology: the specific name is a noun in apposition referring to the species name of the type host.

**Key to species of *Lopharalichus* Gaud & Atyeo, 1996**

1. Both sexes: wide apicoventral spines on genua I, II around base of seta *mG*, much wider than spines on corresponding tibiae I, II; setae *vi* dilated; cuticular spines absent; males with setae *ps*1 roughly triangular with rounded edges (gradually narrowed toward distal end, width of basal part about 4-times wider than distal part); setae *e*2 not bifid basally……. *L. denticulatus* (Mégnin & Trouessart, 1884)

1’. Both sexes: spines on genua I, II about as wide as those on tibiae I, II; setae *vi* not dilated; cuticular spines present on posterolateral margins of opisthosoma; males with setae *ps*1 parallelogram-shaped (width of base subequal to that of distal end); setae *e*2 bifid basally ……. 2

2. In both sexes, setae *si* and *se* piliform, subequal in length (Figure 2C and Figure 3C) ….. *L. beckeri* Mironov et al., 2005

2’ In females, setae *si* always spiculiform; in males, setae *si* either spiculiform or piliform… 3

3. In both sexes, lateral margins of hysterosoma with pronounced spines from level of setae *cp* to *e*2 (Figure 9A and Figure 10A); in males, setae *si* spiculiform, noticeably more robust than *se* (Figure 2E) … *L. spinosus* sp. nov.

3’: In both sexes, spines on the lateral margins of hysterosoma limited to the levels between setae *d*1 to *e*2 (in males), and *d*1 to *f*2 (in females) …. 4

4. In both sexes, solenidion *φIII* longer or equal to the length of genu + tibia III (Figure 14C,H); in females, tips setae *si* not reaching each other …. *L. chiriri* sp. nov.

4’. In both sexes, solenidion *φIII* shorter than the length of genu + tibia III; in females, setae *si* relatively longer, their tips touching each other … 5

5. In both sexes, distance *si:si* about 1.5 longer than distances between *si:se* (Figure 2D and Figure 3D); in males, *si* about twice longer than *se*; in females, setae *si* equal to distance *si:se* ….. *L. tuim* sp. nov.

5’ In both sexes, distance *si:si* approximately equal to the distance *si:se* (Figure 2B and Figure 3B); in males, *si* and *se* subequal in length; in females, setae *si* shorter than the distance between setae *si* and *se* ….. *L. cribriformis* (Mégnin & Trouessart, 1884)

## 4. Discussion

By the time Gaud & Atyeo [5] established the genus *Lopharalichus*, they mentioned that it occurred solely on parrots of the subfamily Aratinginae (sensu Wolters [18]). However, they also referred to this genus as having two undescribed species [5] from parrots then considered in the subfamily Forpinae (sensu Wolters): *Forpus passerinus* and *F. sclateri* (the latter is currently regarded as a subspecies of *Forpus modestus* (Cabanis, 1849)). Herein, a new species is described from the genus *Brotogeris*, previosuly considered in yet another subfamily of Wolters, Brotogerinae. In the current classification of parrots [19], the hosts of *Lopharalichus* are parrots of the family Psittacidae, subfamily Arinae, tribes Arini, Forpini, and Androglossini—it remains to be discovered whether *Lopharalichus* is also present on the tribe Amoropsittacini. Those three tribes account for nearly 140 parrot species (~93% of the arine species), and *Lopharalichus* spp. has been reported from only eight of those hosts so far, including two undescribed species illustrated by Gaud & Atyeo [5].

According to Wright et al. [20], the Arinae—the New World parrots—diverged from the African Psittacinae around the K-T boundary (~66 mya) and diversified approximately 55 mya. *Lopharalichus*, being found only in New World parrots, probably originating between those dates, and given its seemingly uneven distribution on three out of four arine tribes (see above), it probably independently colonized those hosts horizontally rather than vertically. Recent studies have demonstrated that horizontal transfer is an important means of colonizing new hosts e.g., [21,22]. An alternative but less likely scenario would be *Lopharalichus* being present on the arine ancestor and having independently become extinct from several hosts of the tribe Arini (e.g., *Anodorhynchus* Spix, *Cyanopsitta* Bonaparte, *Deroptyus* Wagler, *Diopsittaca* Ridgway, *Enicognathus* Gray, *Leptosittaca* Berlepsch & Stolzmann, *Pionites* Heine, and *Pyrrhura* Bonaparte) and Androglossini (most genera excepting *Brotogeris*, see [19]). In a series of papers, W.T. Atyeo and co-workers investigated the pterolichine feather mites from several of those Arini hosts and did not retrieve any mites that would be later classified in the genus *Lopharalichus* [23,24,25,26,27,28]. Valdebenito et al. [29] examined feather mites from the two species of *Enicognathus* from Chile (also belonging to the Arini) and did not retrieve *Lopharalichus*. As for the tribe Androglossini, only one *Lopharalichus* is known, *L. chiriri* sp. nov. from *Brotogeris chiriri*; the latter tribe contains 10 genera and at least 66 species [14]. Since many of those hosts have not been thoroughly investigated for feather mites, it is reasonable to anticipate that other *Lopharalichus* species may be present in some of those hosts. In the past decade, only a few studies have examined feather mites associated with psittaciform birds in Brazil e.g., [4,30,31,32,33,34]. It is clear, however, that several species remain to be discovered, as nearly 90 parrot species (Psittacidae: Arinae) are found in the country [35].

As in other genera of the *Protolichus* group, the solenidion *σ*1 of genu I in *Lopharalichus* is highly reduced, vestigial, and depending on the position of the specimen on the slide, barely visible. Although the presence of this solenidion was confirmed for some *Lopharalichus* species (e.g., *L. cribriformis*, *L. beckeri*, and *L. spinosus* sp. nov.), it was not possible to confirm its presence in the remaining species studied.

Despite the presence of cuticular spines in the adults, the two examined immature specimens belonging to the species described herein lack such spines. The retention of small cuticular spines on the posterolateral margins of opisthosoma in most adults of *Lopharalichus* species (except in *L. denticulatus*) is not unique to this genus. In other pterolichines belonging to the *Protolichus* generic group, like *Aralichus* Gaud, 1966 and *Distigmesikya* Atyeo, Gaud et Pérez, 1984, the immatures have numerous such spines—in *Aralichus,* they are mostly located caudally, and in *Distigmesikya,* they abundantly cover most of the dorsum) [5,25]. As these mites undergo their final moult to adulthood, those spines disappear in most species. In some of them, however, spines are present in adults, like in both sexes of *Aralichus glaucogularis* Atyeo et Pérez, 1990, and in females of *Scolaralichus vazquezae* Pérez et Atyeo, 1986, *Aralichus menchacai* Pérez et Atyeo, 1989, and *Tanyaralichus elongatus* Pérez et Atyeo, 1989. However, the immatures of the latter two species were not illustrated with cuticular spines [23,24,28].

## 5. Conclusions

With the description of three new species, *Lopharalichus* has effectively doubled its known species count, now encompassing six species: *L. denticulatus* (Mégnin & Trouessart, 1884) (type species), *L. cribriformis* (Mégnin & Trouessart, 1884), *L. beckeri* Mironov et al. 2005, *L. tuim* **sp. nov.**, *L. spinosus* **sp. nov.**, and *L. chiriri* **sp. nov.** However, since most neotropical parrots remain uninvestigated for their feather mites, it is safe to assume that many other *Lopharalichus* species may exist and will eventually be discovered.

## Data Availability

Data are available upon request to the corresponding author.
